# ProteinVolume: calculating molecular van der Waals and void volumes in proteins

**DOI:** 10.1186/s12859-015-0531-2

**Published:** 2015-03-26

**Authors:** Calvin R Chen, George I Makhatadze

**Affiliations:** Department of Biological Sciences and Center for Biotechnology and Interdisciplinary Studies, Rensselaer Polytechnic Institute, 110 8th Street, Troy, NY 12180 USA

**Keywords:** ProteinVolume, Volume calculations, Void volume, van der Waals volume

## Abstract

**Background:**

Voids and cavities in the native protein structure determine the pressure unfolding of proteins. In addition, the volume changes due to the interaction of newly exposed atoms with solvent upon protein unfolding also contribute to the pressure unfolding of proteins. Quantitative understanding of these effects is important for predicting and designing proteins with predefined response to changes in hydrostatic pressure using computational approaches. The molecular surface volume is a useful metric that describes contribution of geometrical volume, which includes van der Waals volume and volume of the voids, to the total volume of a protein in solution, thus isolating the effects of hydration for separate calculations.

**Results:**

We developed ProteinVolume, a highly robust and easy-to-use tool to compute geometric volumes of proteins. ProteinVolume generates the molecular surface of a protein and uses an innovative flood-fill algorithm to calculate the individual components of the molecular surface volume, van der Waals and intramolecular void volumes. ProteinVolume is user friendly and is available as a web-server or a platform-independent command-line version.

**Conclusions:**

ProteinVolume is a highly accurate and fast application to interrogate geometric volumes of proteins. ProteinVolume is a free web server available on http://gmlab.bio.rpi.edu. Free-standing platform-independent Java-based ProteinVolume executable is also freely available at this web site.

**Electronic supplementary material:**

The online version of this article (doi:10.1186/s12859-015-0531-2) contains supplementary material, which is available to authorized users.

## Background

**Figure 1:**
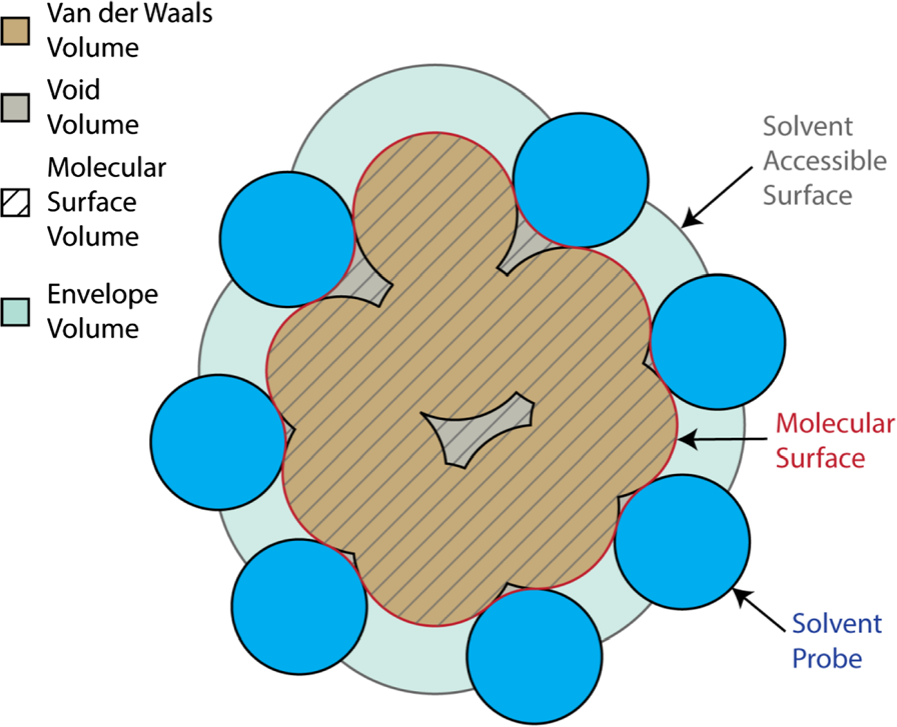
**Schematic diagram depicting surface and volume definitions.** The solvent accessible surface is made by tracing out the center of solvent probes (blue circles) rolled around the entire protein surface. The molecular surface definition cleanly separates geometric or solvent-excluded volume (V_SE_) from the envelope volume (V_E_) reflecting to solute-solvent interactions. The molecular surface volume (V_MS_) is the sum of the van der Waals (V_VDW_) and void volumes (V_Void_).

The volume that a protein occupies in solution is an important thermodynamic parameter: the change in protein volume upon unfolding defines the changes in stability as a function of pressure, ΔV = (∂ΔG/∂P)_T_. Experimental studies have shown that such changes upon unfolding of proteins are small and range from −4.0 to +1.0% [1-3]. The volume of a protein in solution can be divided into its protein-solvent interaction volume and geometric volume. The protein-solvent interaction volume is affected by the hydrophobicity, polarity, and charge distribution of surface residues of the protein. The geometric volume is the solvent-excluded volume, which is enclosed within the solvent-excluded surface (Figure 1). The solvent-excluded surface was termed the molecular surface by Richards in 1977 [4]. In this paper, we will refer to the solvent-excluded volume as the molecular surface volume (V_MS_). The molecular surface volume comprises of the intrinsic volume of protein atoms termed van der Waals volume (V_VDW_), and the intramolecular void volume (V_Void_) that arises due to imperfect packing between protein atoms (Figure 1). The solvent accessible surface is the surface delineated by the center of a solvent probe rolling around the protein. The volume enclosed by this surface is termed the solvent accessible volume (V_SA_). The volume enclosed between the solvent accessible surface and molecular surface is the envelope volume (V_E_ = V_SA_ - V_MS_). It is well established that the voids in the native protein structure determine the pressure unfolding of proteins [5,6]. In this paper, we will focus on the calculation of the geometric volume of a protein enclosed within the molecular surface, which can be computed knowing the Cartesian coordinates of protein atoms found in PDB structure files.

Currently there are several algorithms to calculate geometric volumes of proteins. They can be divided into three distinct categories. The first is 3D grid-based calculations and include VOIDOO [7], AVP [8], 3 V [9], Voronoia [10]. The second category uses analytical methods and includes MSROLL [11], VORLUME [12] and ALPHAVOL [13]. The third category includes calculations based on Delaunay triangulation such as VADAR [14] or Monte Carlo method such as MCVOL [15]. Each of these methods has its own advantages but more importantly some disadvantages. For example, 3D-grid methods have irreproducibility issues due to the positioning of protein structure on the grid. The Delaunay triangulation does perform well in the protein interior but suffers from uncertainty of how protein boundaries are delineated. These issues are sometimes further amplified upon implementation in software packages that are usually written to evaluate a particular property (see comparison in Additional file 1: Table S1).

Several methods calculate V_VDW_ and V_SA_. VOIDOO [7] is a 3D grid-based algorithm that calculates the V_VDW_ and/or V_SA_ of a protein. VORLUME [12] and ALPHAVOL [13] are analytical alpha-shape methods that also calculate V_VDW_ and/or V_SA_. Another method to calculate protein volume involves partitioning the space around each atom into Voronoi polyhedra, as implemented by Finney in 1970 [16] and Richards in 1974 [17]. However, this method does not calculate any of the volumes individually, but instead calculate the sum of the V_VDW_, V_Void_, and portions of the V_E_. Parts of the V_E_ are assigned to surface atoms because the boundary separating protein and bulk solvent is drawn between the surface atoms and neighboring solvent molecules. Thus, the boundary separating protein and bulk solvent is highly dependent on the method used for the placement of the solvent molecules. Depending on the placement method, the volume and packing density of surface atoms will vary. Since parts of the V_E_ are grouped with protein atoms, it is impossible to separate hydration or geometric volume components from the total volume computed using Voronoi polyhedra methods.

It is crucial to separate geometric and hydration volumes of a protein to understand the magnitude of contribution of each of these components to the total volume of a protein in solution. Therefore, it is necessary to calculate the V_MS_ of a protein instead of V_SA_ and V_VDW_. Unfortunately, there are a limited number of non-grid based programs that can calculate V_MS_. MCVOL [15] uses a Monte Carlo algorithm to approximate the V_MS_ of a protein, whereas MSROLL [11] analytically calculates V_MS_. However, both programs have inherent limitations. MCVOL will underestimate V_Void_ when the diameter along the shortest axis of a cavity is larger than 2.8 Å, because a point is considered part of the solvent if it is more than 1.4 Å away from the surface of any protein atom [15]. MSROLL is extremely fast, but it suffers from lower robustness when encountering degenerate geometry. Finally neither is available as a web-server. We present ProteinVolume, a robust method to numerically calculate V_MS_, V_VDW_ and V_Void_ using a flood-fill algorithm to generate the molecular surface and fill the surface interior with high-resolution probes. Volume probes can dynamically reduce their radius when needed, increasing the accuracy of numerical approximation.

## Implementation

ProteinVolume is available as free-standing software as well as via a web-based interface from http://gmlab.bio.rpi.edu. Below we describe the overall properties of the ProteinVolume followed by the description of web-server.

### Surface generation

The surface of a protein is generated from the user provided Protein Data Bank (PDB) coordinates using a flood-fill algorithm operating in the spherical coordinate system, analogous to rolling a ball on the surface of a protein. The furthest atom from the protein center of mass is selected as the starting atom. Then, an exhaustive ray-sphere intersection test is carried out on all angles around the starting atom to find an unoccupied position for a probe with 1.4 Å radius. This is the starting position for the surface algorithm. The starting spherical coordinates are converted into Cartesian coordinates and then the surface is grown from that starting point using a flood-fill algorithm. A hashset is used to store all previously visited locations on each atom surface to prevent backtracking. To detect inter-atom surface probe collisions, all surface probes within nearby spatial bins are tested for distance below a minimum cutoff, the surface probe minimum distance (default value set to 0.1 Å). For reference, this method generates approximately 500,000 surface probes for the native structure of ubiquitin (1UBQ, 76 residues, 1,231 atoms) in ~2 seconds on a single core of an i7-3630QM.

### Volume calculation

**Figure 2:**
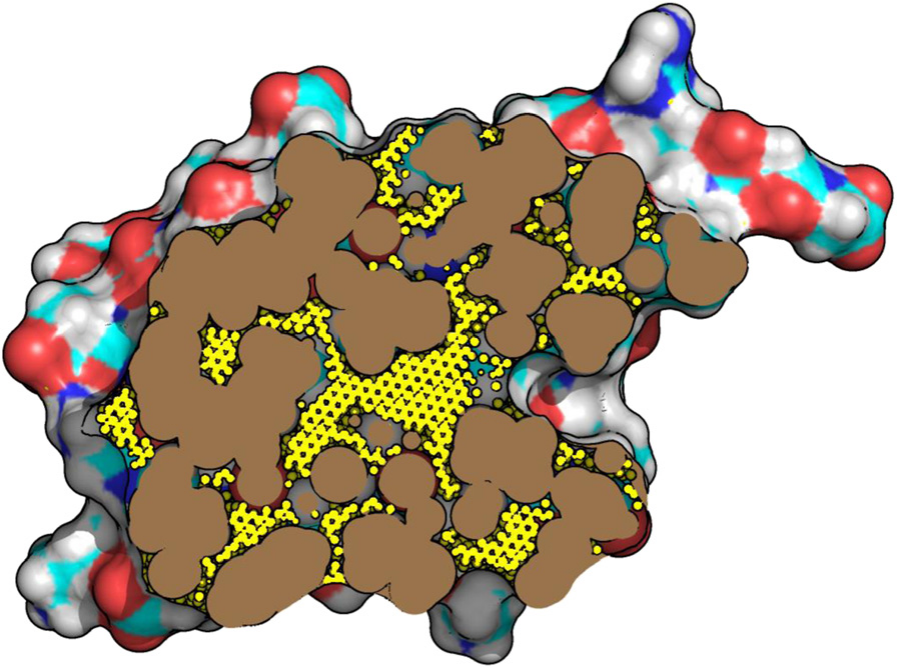
**Cartoon representation of probes filling the voids inside a protein.** For illustrative purposes this picture was generated with the probe size (yellow) fixed at 0.2 Å. Actual calculations were run with the starting probe size of 0.04 Å (see Figure 3).

The total volume and van der Waals volume of a protein is also calculated using a flood-fill algorithm (see Figure 2). The atom closest to the center of mass of the protein is selected as the starting point. A volume probe is then placed at the center of the starting atom and volume probes are grown outwards until they are 1.4 Å away from any surface probe, thus filling the molecular surface. Upon collision with any surface probe, a volume probe is replaced by 8 new volume probes with half its radius as to increase the volume calculation resolution. This process continually repeats itself upon collision until the new volume probe is less than the preset minimum volume probe radius. Volume probes are treated as cubes for the purposes of volume calculations. The sum of all volume probes is calculated and reported as the total protein volume (V_MS_). Van der Waals volume is also calculated during the same step as the total volume calculation procedure, but with an additional check of whether the volume probe is within the van der Waals radius of a protein atom. A probe which lies on top of a van der Waals boundary will be randomly accepted based on its magnitude of overlap with the atom. This increases the accuracy of the van der Waals volume calculation and reduces the volume underestimation of numerical integration methods. The sum of all van der Waals volume probes is calculated and reported as van der Waals protein volume (V_VDW_). Void volume, V_Void_, is calculated as the difference between the total volume and the van der Waals volume.

### Optimizations

Grid-based spatial binning is employed to reduce the number of collision checks when placing a new volume probe in the protein. The entire 3D coordinate space is divided into cubic spatial bins of 2 Å diameter. This value is slightly larger than the radius of the largest protein atom which will minimize the number of possible bins an atom can occupy. Each existing protein atom and generated surface probe is added into a hashmap of spatial bins before volume calculation. The data structure of the hashmap is a spatial bin index and an ArrayList of atoms/probes. The spatial bin index is calculated from the 9 possible extreme edges of each sphere and duplicate bin indices are ignored. When testing for a collision between volume probes and surface atoms or nearby protein atoms, only spatial bins surrounding the volume probe are selected for collision testing as to reduce computational time. This results in an overall runtime complexity of O(n), where n is the number of atoms in the system.

### Language and libraries

ProteinVolume was programmed in Java (JDK 1.7) using the Trove collections library for higher performance and overall lower memory usage. ProteinVolume is platform independent and can be run on any platform with a Java runtime environment.

### ProteinVolume web interface

ProteinVolume web interface allows users to upload PDB files and run ProteinVolume from any device without expending their local computing resources. We have strived to create a clean, user-friendly, and responsive interface for ease of use. All interactions with the server are AJAX-powered, which provides a native feel to the application. Users are presented with a form that allows them to upload file(s) of interest and fill in their names and email addresses. Anonymous users are allowed to upload one PDB file whereas users providing their name are allowed to upload up to ten PDB files. After the PDB files are uploaded, users are placed into a queue. As resources become available, the job is executed and the output of the program is displayed in real time to the user and a progress bar is displayed. The progress bar shows the percent completion value, estimated based on the total number of atoms in all submitted PDB files and the selected ProteinVolume options.

### Input structure preparation

The default option of ProteinVolume uses explicit hydrogen atoms and Bondi [18] van der Waals radii for all atoms due to overestimation of van der Waals volumes when united atom radii are used. It is highly recommended to energy minimize all structures before volume processing to reduce unfavorable steric clashes that will skew volume results and make volume comparisons inaccurate. For example, we routinely energy minimize our proteins using the CHARMM27 [19] all-atom forcefield in GROMACS [20] for 1 ps using the steepest decent method in implicit solvent and a 1 nm cutoff for electrostatic interactions. This will also add all hydrogen atoms to the structure. The user can add minimization as a preprocessing option to web server calculations. Alternatively, the hydrogen atoms can be explicitly [12] modeled using REDUCE software [21]. In the executable version of ProteinVolume, the user can modify the van der Waals radii set by editing parameter file. If hydrogen atom radius is set to zero, hydrogens will be ignored in the calculations.

### Performance

The volume calculation of a protein ranges from seconds to minutes depending on protein size and program options. On a single core of an i7-3630QM @ 2.4ghz, the structure of ubiquitin (1UBQ, 76 residues) takes ~1 minute to calculate with 0.08 Å starting probe size, 0.02 Å ending probe size, and 0.1 Å surface probe minimum distance. With the current server hardware the same protein with the same parameter settings takes ~9 min. The computational complexity of the algorithm is O(n) or linear, where n is the number of atoms in the system, due to spatial binning optimizations which limit the number of pairwise distance calculations.

### Robustness

A set of 1,379 high-resolution (<1.7 Å) crystal structures had their native ensembles modeled and calculated with ProteinVolume. MODELLER [22] was used to model the native ensemble, which contained 11 structures per protein. The range of protein sizes was between 40 to 1,052 amino acid residues. The total number of structures tested was 15,169. For all structures, ProteinVolume successfully calculated volumes without runtime errors.

## Results

### The effects of the probe size parameters

Three parameters, starting probe size, ending probe size, and surface probe minimum distance, have a significant effect on the running time and accuracy of the algorithm.

**Figure 3:**
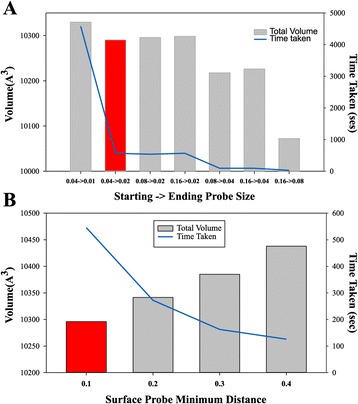
**The effects of the probe size parameters on the running time and accuracy of the algorithm.** Panel **A**. Dependence on the starting probe size. Panel **B**. Dependence on the surface probe minimum distance. Blues lines – the running time; Red bars – default parameters used in all calculations (see text for details).

The starting probe size is the initial radius of probes prior to collision with protein atoms or surface probes. Probes halve in radius upon collision with protein atoms or surface probes to increase the accuracy of calculations. The ending probe size specifies the minimum radius of all probes. Probes that would become smaller than the ending probe size after division are prevented from dividing. Increasing the starting and ending probe sizes speeds up computational time at the expense of volume accuracy due to imperfect packing of the probes around the edges of protein atoms and the protein surface. The default value of starting and ending probe sizes is 0.08 Å and 0.02 Å, respectively, which provides a good balance between runtime and accuracy (see Figure 3A).

The surface probe minimum distance is the minimum distance at which two surface probes can be placed next to each other. When this value is increased, surface probe density decreases which causes a significant reduction in pairwise distance calculations made and reduces processing time taken. The default value for surface resolution is 0.1 Å. Increasing this up to 0.4 Å will decrease computational time at the expense of accuracy of the calculations (see Figure 3B). A surface probe minimum distance of 0.1 Å generates a very high-resolution surface of approximately 5,000 probes per a single isolated atom.

### Benchmarking

ProteinVolume was benchmarked against two volume calculation programs: MCVOL [15] and MSROLL [11]. MCVOL uses a Monte Carlo algorithm to approximate the V_MS_ and V_Void_ of a protein. MSROLL analytically calculates the V_MS_ of a protein. Triangles occupying the intersection volume between atoms are discarded. V_MS_ is calculated by summing the volume of each triangular pyramid formed by the tessellated surface to the center of each atom. 217 ultra-high resolution (0.7-1.2 Å) crystal structures [23,24] were selected for benchmarking volume calculations Additional file 2. Ultra High Resolution Protein Set (0.73 - 1.20 Å). These two programs were selected because they directly compute V_MS_. The average V_MS_ deviation between ProteinVolume and MCVOL or MSROLL was 0.2% and 0.7%, respectively (Additional file 3: Figure S1). The excellent agreement of ProteinVolume, with MSROLL and MCVOL shows that ProteinVolume is accurately calculating V_MS_. Since VOIDOO, Vorlume, and AlphaVol directly compute V_SA_ instead of V_MS_, direct comparison with ProteinVolume volumes is not possible, yet the V_VDW_ computed by for example VOIDOO is in excellent agreement with V_VDW_ computed by ProteinVolume (see Additional file 3: Figure S1). To test whether ProteinVolume accuracy was dependent on crystallographic resolution, calculations performed on a set of proteins, solved to an ultra-high resolution (0.7 - 1.2 Å, n = 217) was compared to a set solved to high resolution (1.2 - 1.7 Å, n = 1,161). As expected [25], both sets display the same slope and intercept for the dependence of volume on the protein size (Additional file 3: Figure S1). This indicates that accuracy of ProteinVolume is independent of the crystallographic resolution.

### Scaling behavior of geometric volumes of proteins

**Figure 4:**
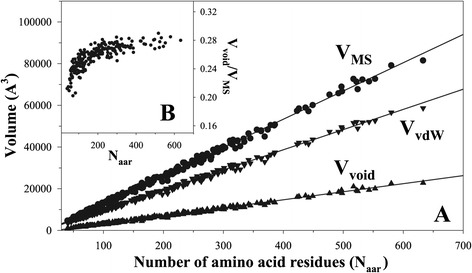
**The size scaling behavior of geometric of volumes of proteins.** Panel **A**. Dependence of the molecular surface volume (circles, V_MS_), the van der Waals volume (triangles, V_VDW_) and void volumes (upside-down triangles, V_Void_) on number of amino acid residues in proteins (N_aar_). Panel **B**. Dependence of fraction of void volume on protein size.

Figure 4A compares the dependences of V_MS_, V_VDW_ and V_Void_ on the number of amino acid residues in proteins. The dependence is linear in all three cases suggesting that as the protein size increases, the corresponding geometric volumes also increase. The slopes of the dependences, however is not the same, it is the largest for V_MS_ and smallest for V_Void_. In other words, as the total volume of protein increases the fraction of void volume, f_Void_ = V_Void_/V_MS_ increases nonlinearly. In fact smaller proteins have smaller f_Void_ than the larger ones (Figure 4B). The dependence plateaus at ~200-250 amino acid residues which is considered to be an upper limit of protein domains [26,27]. Longer proteins with more than ~250 amino acid residues usually consist of multiple structural domains [28].

The void volumes inside the proteins, i.e. the magnitude of V_Void_, have been implicated in determining the pressure unfolding of proteins [5,6]. The prediction based on the scaling behavior of V_Void_ is that larger proteins will be more prone to unfold under pressure. This prediction still awaits experimental validation.

## Conclusions

We present ProteinVolume, a volume calculator that reports the van der Waals (V_VDW_), void (V_Void_), and total volume (V_MS_) enclosed within the molecular surface a protein. The V_MS_, or solvent-excluded volume, can be thought of as the geometric volume contribution of a protein which consists of van der Waals and intramolecular void volume. This allows us to clearly separate the volume contribution of the protein geometry (V_MS_) and the protein-solvent interactions (hydration volume). The sum of these two components should result in a better approximation of the apparent volume of a protein molecule in solution than other computational models which are based on the volume enclosed by the accessible surface area. Finally, partitioning the volume components into geometric (V_MS_) and hydration components will lead to a quantitative insight of each term, and will allow rational engineering of volume changes in proteins.

## Availability and requirements

**Project name**: ProteinVolume

**Project home page**: http://gmlab.bio.rpi.edu

**Operating system(s):** Platform independent

**Programming language**: Java

**Other requirements**: Java Runtime Environment 1.7 and above

**License**: Closed source proprietary

**Any restrictions to use by non-academics**: none

## Additional files

Additional file 1: Table S1. Comparison of Different Software Packages for Calculation of Volumes of Proteins. Additional file 2:
**Ultra High Resolution Protein Set (0.73 - 1.20 Å).**
Additional file 3: Figure S1. The size scaling behavior of geometric of volumes of proteins and comparison of the volumes calculated using ProteinVolume with other software packages. Panel A. Dependence of the molecular surface volume (circles, V_MS_), the van der Waals volume (triangles, V_VDW_) and void volumes (upside-down triangles, V_Void_) on number of amino acid residues in proteins (N_aar_) from ultra-high crystallographic resolution (0.7-1.2 Å) set (red symbols) and high crystallographic resolution (1.2-1.7 Å) set (open symbols) calculated using ProteinVolume. The linear regression lines for ProteinVolume calculations on ultra-high and high resolution sets are indistinguishable, indicating that ProteinVolume results are not dependent on crystallographic resolution. The results from ProteinVolume are also compared to relevant volumes calculated using McVol (blue squares) and MSROLL (green triangles). The van der Waals (V_VDW_) volumes calculated by VOIDOO are shown in cyan circles. Panel B. Dependence of fraction of void volume on protein size for ultra-high crystallographic resolution (0.7-1.2 Å) set (red circles) and high crystallographic resolution (1.2-1.7 Å) set (open squares) calculated using ProteinVolume.

## References

[1] Royer CA (2002). Revisiting volume changes in pressure-induced protein unfolding. Biochim Biophys Acta.

[2] Chalikian TV (2008). On the molecular origins of volumetric data. J Phys Chem B.

[3] Schweiker KL, Fitz VW, Makhatadze GI (2009). Universal convergence of the specific volume changes of globular proteins upon unfolding. Biochemistry.

[4] Richards FM (1977). Areas, volumes, packing and protein structure. Annu Rev Biophys Bioeng.

[5] Frye KJ, Royer CA (1998). Probing the contribution of internal cavities to the volume change of protein unfolding under pressure. Protein Sci.

[6] Roche J, Caro JA, Norberto DR, Barthe P, Roumestand C, Schlessman JL (2012). Cavities determine the pressure unfolding of proteins. Proc Natl Acad Sci U S A.

[7] Kleywegt GJ, Jones TA (1994). Detection, delineation, measurement and display of cavities in macromolecular structures. Acta Crystallogr D Biol Crystallogr.

[8] Cuff AL, Martin AC (2004). Analysis of void volumes in proteins and application to stability of the p53 tumour suppressor protein. J Mol Biol.

[9] Voss NR, Gerstein M (2010). 3V: cavity, channel and cleft volume calculator and extractor. Nucleic Acids Res.

[10] Rother K, Hildebrand PW, Goede A, Gruening B, Preissner R (2009). Voronoia: analyzing packing in protein structures. Nucleic Acids Res.

[11] Connolly ML (1985). Computation of Molecular Volume. J Am Chem Soc.

[12] Cazals F, Kanhere H, Loriot S (2011). Computing the Volume of a Union of Balls: A Certified Algorithm. ACM.

[13] Edelsbrunner H, Koehl P (2003). The weighted-volume derivative of a space-filling diagram. Proc Natl Acad Sci U S A.

[14] Willard L, Ranjan A, Zhang H, Monzavi H, Boyko RF, Sykes BD (2003). VADAR: a web server for quantitative evaluation of protein structure quality. Nucleic Acids Res.

[15] Till MS, Ullmann GM (2010). McVol - A program for calculating protein volumes and identifying cavities by a Monte Carlo algorithm. J Mol Model.

[16] Finney JL (1970). Random Packings and Structure of Simple Liquids.1. Geometry of Random Close Packing. Proc R Soc Lon Ser-A.

[17] Richards FM (1974). Interpretation of Protein Structures - Total Volume, Group Volume Distributions and Packing Density. J Mol Biol.

[18] Bondi A (1964). Van Der Waals Volumes + Radii. J Phys Chem-Us.

[19] Brooks BR, Brooks CL, Mackerell AD, Nilsson L, Petrella RJ, Roux B (2009). CHARMM: The Biomolecular Simulation Program. J Comput Chem.

[20] Pronk S, Pall S, Schulz R, Larsson P, Bjelkmar P, Apostolov R (2013). GROMACS 4.5: a high-throughput and highly parallel open source molecular simulation toolkit. Bioinformatics.

[21] Word JM, Lovell SC, Richardson JS, Richardson DC (1999). Asparagine and glutamine: using hydrogen atom contacts in the choice of side-chain amide orientation. J Mol Biol.

[22] Fiser A, Sali A (2003). MODELLER: Generation and refinement of homology-based protein structure models. Method Enzymol.

[23] Bush J, Makhatadze GI (2011). Statistical analysis of protein structures suggests that buried ionizable residues in proteins are hydrogen bonded or form salt bridges. Proteins.

[24] Loladze VV, Makhatadze GI (2011). Energetics of charge-charge interactions between residues adjacent in sequence. Proteins.

[25] Fleming PJ, Richards FM (2000). Protein packing: Dependence on protein size, secondary structure and amino acid composition. J Mol Biol.

[26] Trifonov EN, Berezovsky IN (2003). Evolutionary aspects of protein structure and folding. Curr Opin Struct Biol.

[27] Sandhya S, Rani SS, Pankaj B, Govind MK, Offmann B, Srinivasan N (2009). Length variations amongst protein domain superfamilies and consequences on structure and function. PLoS One.

[28] Privalov PL (1982). Stability of proteins. Proteins which do not present a single cooperative system. Adv Protein Chem.

