# Supplementary material for: ProteinVolume: calculating molecular van der Waals and void volumes in proteins
Source: BMC Bioinformatics. 2015 Mar 26;16(1):101. doi: 10.1186/s12859-015-0531-2 (PMC4379742; doi:10.1186/s12859-015-0531-2)
Supplement: Additional file 1: Table S1. — Comparison of Different Software Packages for Calculation of Volumes of Proteins. [file 12859_2015_531_MOESM1_ESM.pdf]

TABLE S1 Comparison of Different Software Packages for Calculation of Volumes of Proteins

**Software**	**Volume Type**	**Web-Server**	**vdW radii**	**vdW radii selectable**	**Explicit Hydrogens**	**Probe size adjustable**	**Algorithm**	**Issues**
ProteinVolume	V_{MS} V_{Void} V_{VDW}	Yes [a]	All-atom	Yes	Yes	Yes	Flood-Filling	
MCVOL [1]	V_{MS} V_{VDW} V_{Void}	No	All-atom	Yes	Yes	Yes	Monte Carlo	Slight deviation per run (<0.5%)
VOIDOO [2]	V_{Vdw} V_{SA} V_{Void}	No	All-atom	Yes	Yes	Yes	3D-grid	No V_{MS}
AVP [3]	V_{Void}	No	United	No	Yes	Yes	3D-grid	No V_{MS} AVP produces V_{Void} volumes are 30% lower than all other programs.
[3]V [4]	V_{MS} V_{VDW} V_{Void}	Yes [b]	All-atom	Yes (not for web-server)	Yes	Yes	3D-grid	Web server has a max grid resolution of 0.5A, which produces V_{MS} that is 10-15% larger than other software.
Voronoia [5]	V_{MS} V_{Void} V_{VDW}	Yes [c]	United	No [e]	No	Yes	3D-grid	Large overestimation of V_{MS} due to radii set. Some heavy atoms missing from volume calculation.
VADAR [6]	V_{MS} + parts of V_{E}	Yes [d]	United	No [f]	No	No	Voronoi/ Delaney	No V_{MS}
GEPOL [7]	V_{MS} V_{VDW}	No	All-atom	Yes	Yes	N/A	Numerical tessellation	Limit to 100,000 surface points
MSROLL [8]	V_{MS} V_{VDW} V_{Void}	No	All-atom	Yes	Yes	N/A	Analytical	Susceptible to geometrical degeneracies
VORLUME [9]	V_{Vdw} V_{SA}	No	United	No	No	N/A	Alpha-Shapes	No V_{MS}
ALPHAVOL [10]	V_{Vdw}, V_{SA}	No	United	No	Polar Only	N/A	Alpha-Shapes	No V_{MS} , limit 500 amino acid residues

a ProteinVolume - gmlab.bio.rpi.edu
b 3V - http://3vee.molmovdb.org/volumeCalc.php
c Voronoia - http://bioinformatics.charite.de/voronoia/index.php?site=input
d VADAR - http://vadar.wishartlab.com/
e selectable from ProtOr, or Stouten
f selectable from Chothia, Eisenberg, Richards or Shrake.

References

1. Till MS, Ullmann GM: **McVol - A program for calculating protein volumes and identifying cavities by a Monte Carlo algorithm.** *J Mol Model* 2010, **16:**419-429.
2. Kleywegt GJ, Jones TA: **Detection, delineation, measurement and display of cavities in macromolecular structures.** *Acta crystallographica Section D, Biological crystallography* 1994, **50:**178-185.
3. Cuff AL, Martin AC: **Analysis of void volumes in proteins and application to stability of the p53 tumour suppressor protein.** *Journal of molecular biology* 2004, **344:**1199-1209.
4. Voss NR, Gerstein M: **3V: cavity, channel and cleft volume calculator and extractor.** *Nucleic Acids Res* 2010, **38:**W555-562.
5. Rother K, Hildebrand PW, Goede A, Gruening B, Preissner R: **Voronoia: analyzing packing in protein structures.** *Nucleic Acids Res* 2009, **37:**D393-395.
6. Willard L, Ranjan A, Zhang H, Monzavi H, Boyko RF, Sykes BD, Wishart DS: **VADAR: a web server for quantitative evaluation of protein structure quality.** *Nucleic Acids Res* 2003, **31:**3316-3319.
7. Silla E, Tunon I, Pascualahuir JL: **Gepol - an Improved Description of Molecular-Surfaces .2. Computing the Molecular Area and Volume.** *J Comput Chem* 1991, **12:**1077-1088.
8. Connolly ML: **Computation of Molecular Volume.** *J Am Chem Soc* 1985, **107:**1118-1124.
9. Cazals F, Kanhere H, Loriot S: **Computing the Volume of a Union of Balls: A Certified Algorithm.** *Acm T Math Software* 2011, **38:**1-25.
10. Edelsbrunner H, Koehl P: **The weighted-volume derivative of a space-filling diagram.** *P Natl Acad Sci USA* 2003, **100:**2203-2208.
