# Supplementary material for: ProteinVolume: calculating molecular van der Waals and void volumes in proteins
Source: BMC Bioinformatics. 2015 Mar 26;16(1):101. doi: 10.1186/s12859-015-0531-2 (PMC4379742; doi:10.1186/s12859-015-0531-2)
Supplement: Additional file 2: — Ultra High Resolution Protein Set (0.73 - 1.20 Å). [file 12859_2015_531_MOESM3_ESM.pdf]

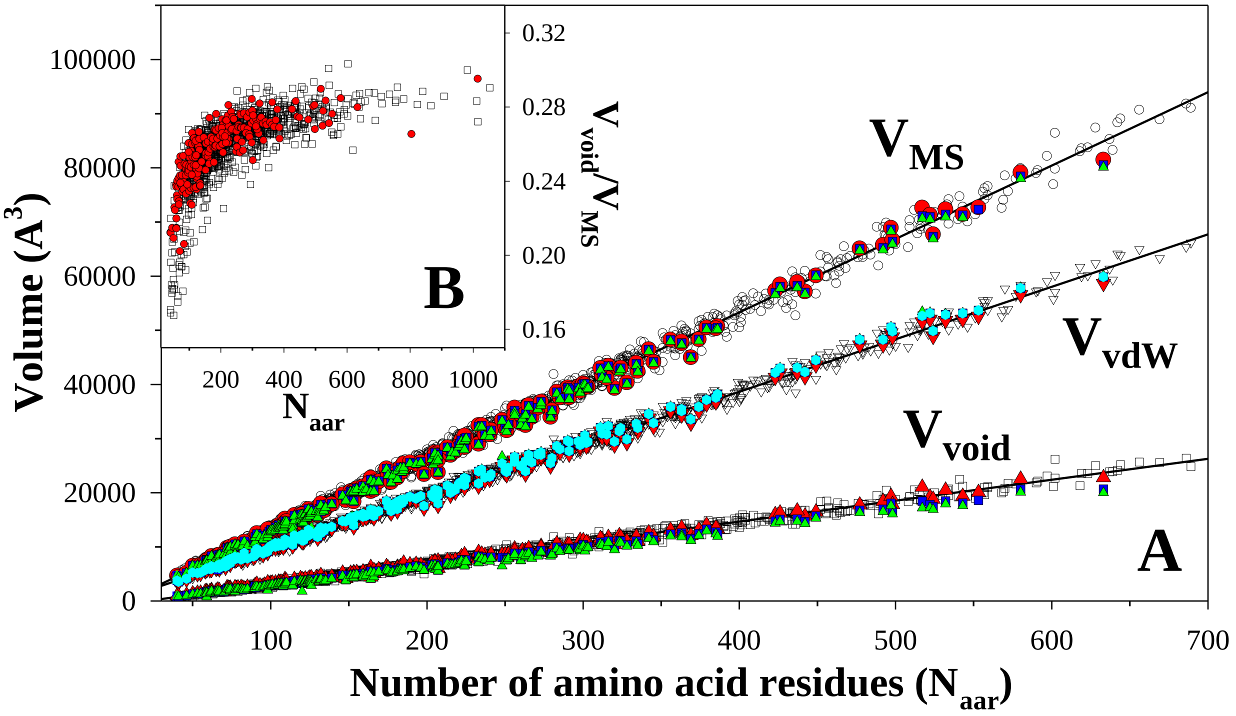

Figure S1. The size scaling behavior of geometric of volumes of proteins and comparison of the volumes calculated using ProteinVolume with other software packages. ***Panel A.*** Dependence of the molecular surface volume (circles, V_{MS}), the van der Waals volume (triangles, V_{VDW}) and void volumes (upside-down triangles, V_{Void}) on number of amino acid residues in proteins (N_{aar}) from ultra-high crystallographic resolution (0.7-1.2 Å) set (red symbols) and high crystallographic resolution (1.2-1.7 Å) set (open symbols) calculated using ProteinVolume. The linear regression lines for ProteinVolume calculations on ultra-high and high resolution sets are indistinguishable, indicating that ProteinVolume results are not dependent on crystallographic resolution. The results from ProteinVolume are also compared to relevant volumes calculated using McVol (blue squares) and MSROLL (green triangles). The van der Waals (V_{VDW}) volumes calculated by VOIDOO are shown in cyan circles. ***Panel B.*** Dependence of fraction of void volume on protein size for ulta-high crystallographic resolution (0.7-1.2 Å) set (red circles) and high crystallographic resolution (1.2-1.7 Å) set (open squares) calculated using ProteinVolume.
