# Supplementary material for: ProteinVolume: calculating molecular van der Waals and void volumes in proteins
Source: BMC Bioinformatics. 2015 Mar 26;16(1):101. doi: 10.1186/s12859-015-0531-2 (PMC4379742; doi:10.1186/s12859-015-0531-2)
Supplement: Additional file 3: Figure S1. — The size scaling behavior of geometric of volumes of proteins and comparison of the volumes calculated using ProteinVolume with other software packages. Panel A. Dependence of the molecular surface volume (circles, VMS), the van der Waals volume (triangles, VVDW) and void volumes (upside-down triangles, VVoid) on number of amino acid residues in proteins (Naar) from ultra-high crystallographic resolution (0.7-1.2 Å) set (red symbols) and high crystallographic resolution (1.2-1.7 Å) set (open symbols) calculated using ProteinVolume. The linear regression lines for ProteinVolume calculations on ultra-high and high resolution sets are indistinguishable, indicating that ProteinVolume results are not dependent on crystallographic resolution. The results from ProteinVolume are also compared to relevant volumes calculated using McVol (blue squares) and MSROLL (green triangles). The van der Waals (VVDW) volumes calculated by VOIDOO are shown in cyan circles. Panel B. Dependence of fraction of void volume on protein size for ultra-high crystallographic resolution (0.7-1.2 Å) set (red circles) and high crystallographic resolution (1.2-1.7 Å) set (open squares) calculated using ProteinVolume. [file 12859_2015_531_MOESM2_ESM.pdf]

Ultra High Resolution Protein Set (0.73 - 1.20 Å)

First 4 letters are PDB code, fifth letter is chain id. In parenthesis - number of amino acid residues and crystallographic resolution in Å.

2ERL_ (40, 1.00); **1P9GA (**41, 0.84); **1CNR_ (**46, 1.05); **2A26A (**50, 1.20); **1BRFA (**53, 0.95); **2CS7C (**55, 1.20); **1G6XA (**58, 0.86); **1OAIA (**59, 1.00); **2FMAA (**59, 0.85); **2G6FX (**59, 0.92); **1NKD_ (**59, 1.07); **2IGD_ (**61, 1.10); **1G2BA (**62, 1.12); **1V6PA (**62, 0.87); **2SN3_ (**65, 1.20); **1C9OA (**66, 1.17); **1HG7A (**66, 1.15); **1TUKA (**67, 1.12); **1VFYA (**67, 1.15); **2DLBA (**70, 1.20); **2B97A (**71, 0.75); **1WM3A (**72, 1.20); **1WXCB (**72, 1.20); **1CC8A (**73, 1.02); **1I27A (**73, 1.02); **1L9LA (**74, 0.92); **1OK0A (**74, 0.93); **2BWFB (**77, 1.15); **1USMA (**77, 1.20); **1UCRB (**78, 1.20); **1XMKA (**79, 0.97); **1IQZA (**81, 0.92); **1R6JA (**82, 0.73); **1ZZKA (**82, 0.95); **2D8DB (**83, 1.15); **1B0YA (**85, 0.93); **1CTJ_ (**89, 1.10); **1U07A (**90, 1.13); **2BT9A (**90, 0.94); **1X6IB (**91, 1.20); **2FHZB (**93, 1.15); **1C5EA (**95, 1.10); **1LNIB (**96, 1.00); **1CZPA (**98, 1.17); **2AIBA (**98, 1.10); **1NQJA (**98, 1.00); **1KZKB (**99, 1.09); **1MN8D (**100, 1.00); **1PSRB (**100, 1.05); **1M2DA (**101, 1.05); **2DKOB (**103, 1.06); **2H3LA (**103, 1.00); **1LKKA (**105, 1.00); **1TQGA (**105, 0.98); **2GBAA (**105, 0.92); **1M9ZA (**105, 1.05); **2FRGP (**106, 1.19); **1V8HA (**107, 1.20); **1GMXA (**108, 1.10); **1J0PA (**108, 0.91); **2AGYD (**108, 1.10); **1BKRA (**109, 1.10); **1RWYA (**109, 1.05); **2FHZA (**109, 1.15); **1H4XA (**110, 1.16); **1I8OA (**114, 1.15); **2CHHA (**114, 1.00); **1F86A (**115, 1.10); **1SAUA (**115, 1.12); **1O7IA (**119, 1.20); **2ICCA (**119, 1.20); **2F01B (**120, 0.85); **1W0NA (**120, 0.80); **1VR7A (**120, 1.20); **1WN2A (**121, 1.20); **2GUDB (**121, 0.94); **1LWBA (**122, 1.05); **2FWGA (**122, 1.10); **1VL9A (**123, 0.97); **1DY5A (**123, 0.87); **1GU2A (**124, 1.19); **1UNQA (**124, 0.98); **1NWZA (**125, 0.82); **2FJ8A (**125, 1.19); **1VZIA (**126, 1.15); **1JBEA (**126, 1.08); **4LZT_ (**129, 0.95); **1KNLA (**130, 1.20); **1JF8A (**131, 1.12); **1OH0B (**131, 1.10); **1C7KA (**132, 1.00); **1IFC_ (**132, 1.19); **1TU9A (**134, 1.20); **2AXWA (**134, 1.05); **1NKIA (**135, 0.95); **1CZ9A (**139, 1.20); **1RG8A (**146, 1.10); **1EXRA (**148, 1.00); **1A6M_ (**151, 1.00); **1QTNA (**152, 1.20); **1GWMA (**153, 1.15); **2C9VA (**153, 1.07); **1J98A (**153, 1.20); **2FLHB (**155, 1.20); **1UOWA (**159, 1.04); **1Y93A (**159, 1.03); **1P6OB (**161, 1.14); **1L3KA (**163, 1.10); **1TT8A (**164, 1.00); **1WKQA (**164, 1.17); **1N62A (**166, 1.09); **2CE2X (**166, 1.00); **1OBOA (**169, 1.20); **1AMM_ (**174, 1.20); **2AU7A (**175, 1.05); **1EB6A (**177, 1.00); **2C2UA (**178, 1.10); **1I4UA (**181, 1.15); **1WC2A (**181, 1.20); **1KT6A (**183, 1.10); **2AT7X (**184, 0.98); **1PMHX (**185, 1.06); **1QV0A (**185, 1.10); **2BBRA (**189, 1.20); **2PTH_ (**193, 1.20); **2CARA (**196, 1.09); **1QQ4A (**198, 1.20); **1Z0WA (**203, 1.20); **1JM1A (**204, 1.11); **1IXBA (**205, 0.90); **2AB0A (**205, 1.10); **2C71A (**205, 1.05); **1HDOA (**206, 1.15); **1G66A (**207, 0.90); **1H4GB (**207, 1.10); **1KWNA (**207, 1.20); **1SFSA (**213, 1.07); **1ME3A (**215, 1.20); **1K4IA (**216, 0.98); **1W66A (**218, 1.08); **1FYEA (**220, 1.20); **1O08A (**221, 1.20); **2A6ZA (**222, 1.00); **1OLRA (**224, 1.20); **1UAIA (**224, 1.20); **2AWKA (**224, 1.15); **1KG2A (**225, 1.20); **1FSGC (**233, 1.05); **1K7CA (**233, 1.12); **1YMTA (**235, 1.20); **1JBC_ (**237, 1.20); **1GVKB (**240, 0.94); **1QL0A (**241, 1.10); **1HBNC (**248, 1.16); **2J27A (**250, 1.15); **1ZJYA (**251, 1.05); **1QXYA (**252, 1.04); **1XQOA (**256, 1.03); **1MOOA (**256, 1.05); **1P1XA (**260, 0.99); **1UWCA (**261, 1.08); **1NYMA (**263, 1.20); **1ARB_ (**263, 1.20); **1XDNA (**265, 1.20); **1WUIS (**267, 1.04); **1KQPA (**271, 1.03); **2CI1A (**273, 1.08); **1WXCA (**273, 1.20); **1IC6A (**279, 0.98); **2BOGX (**280, 1.04); **1E9GB (**283, 1.15); **1QTWA (**285, 1.02); **1LC0A (**290, 1.20); **2EUTA (**291, 1.12); **1RTQA (**291, 0.95); **2J45B (**297, 1.14); **2CIWA (**298, 1.15); **2BLNA (**298, 1.20); **8A3HA (**300, 0.97); **2CNQA (**301, 1.00); **1V0LA (**302, 0.98); **1ZL0B (**303, 1.10); **1Z2NX (**311, 1.20); **2IAVA (**312, 1.07); **1T2DA (**315, 1.10); **1PWMA (**316, 0.92); **1YS1X (**320, 1.10); **1DS1A (**323, 1.08); **1RYOA (**324, 1.20); **1OEWA (**328, 0.90); **2BW4A (**334,

0.90); **2C1VA (**335, 1.20); **1YFQA (**342, 1.10); **1YQSA (**345, 1.05); **1M15A (**356, 1.20); **1C0PA (**363, 1.20); **1VYRA (**363, 0.90); **1GA6A (**369, 1.00); **1N8KA (**374, 1.13); **3SIL_ (**379, 1.05); **1KJQB (**385, 1.05); **1MUWA (**386, 0.86); **1RA0A (**423, 1.12); **1UG6A (**426, 0.99); **2BMOA (**437, 1.20); **1HBNB (**442, 1.16); **2BF6A (**449, 0.97); **1M1NA (**477, 1.16); **2FBAA (**492, 1.10); **1QW9A (**497, 1.20); **1GWEA (**498, 0.88); **1JETA (**517, 1.20); **1M1NB (**522, 1.16); **1Q6ZA (**524, 1.00); **1WUIL (**532, 1.04); **1HBNA (**543, 1.16); **1UWKB (**553, 1.19); **2BHUA (**580, 1.10); **1SU8A (**633, 1.10); **1N62B (**804, 1.09); **1QWNA (**1014, 1.20).

High Resolution Protein Set (1.20 – 1.70 Å).

First 4 letters are PDB code, fifth letter is chain id. In parenthesis - number of amino acid residues and crystallographic resolution in Å.

1JEKA (40, 1.50); **2FP7A (**40, 1.68); **2IZXB (**41, 1.30); **1FD3A (**41, 1.35); **1HTRP (**43, 1.62); **1CZQA (**45, 1.50); **1P3QQ (**45, 1.70); **2BEQD (**46, 1.60); **1N13A (**46, 1.40); **1M93A (**46, 1.65); **1JM0A (**48, 1.70); **1UFIA (**48, 1.65); **1IRQB (**49, 1.50); **1JCDA (**50, 1.30); **1JY2O (**51, 1.40); **1UPTH (**51, 1.70); **1GVDA (**52, 1.45); **1RH6B (**52, 1.70); **1Y4MA (**53, 1.60); **2ERWA (**53, 1.40); **2BAYE (**59, 1.50); **1KQ1A (**60, 1.55); **1N7SA (**63, 1.45); **1BF4A (**63, 1.60); **1WT6D (**63, 1.60); **1TO2I (**64, 1.30); **2CC6A (**64, 1.27); **2A50A (**64, 1.30); **1YU5X (**67, 1.40); **1FR3A (**67, 1.50); **1WU9B (**67, 1.54); **1A7W_ (**68, 1.55); **2G7OA (**68, 1.40); **1J2LA (**68, 1.70); **1X2IA (**68, 1.45); **2AD6B (**69, 1.50); **1C48A (**69, 1.60); **1ZXTA (**69, 1.70); **1O82A (**70, 1.46); **1A8O_ (**70, 1.70); **2CZSA (**70, 1.50); **1IHRB (**74, 1.55); **1QX2A (**75, 1.44); **1QGWA (**75, 1.63); **1UB4C (**75, 1.70); **1V5IB (**76, 1.50); **1DP7P (**76, 1.50); **2AYDA (**76, 1.60); **2B5AD (**76, 1.54); **1UV7A (**76, 1.70); **2H7ZB (**77, 1.50); **1VCC_ (**77, 1.60); **1PBYC (**78, 1.70); **1TH7A (**78, 1.68); **1N7SC (**79, 1.45); **1ZS4A (**82, 1.70); **1ZS4B (**82, 1.70); **1EZGA (**82, 1.40); **2O30A (**82, 1.66); **1I71A (**83, 1.45); **2CVIA (**83, 1.50); **1UGIA (**83, 1.55); **1FR2A (**83, 1.60); **1ZEQX (**84, 1.50); **2BOPA (**85, 1.70); **1JI7C (**85, 1.45); **1YO3A (**85, 1.65); **2FQ3A (**85, 1.40); **1T0PB (**86, 1.66); **2CB8A (**86, 1.40); **2BKYX (**86, 1.70); **1NA3A (**86, 1.55); **1GVP_ (**87, 1.60); **2I6VA (**87, 1.63); **1LLMC (**87, 1.50); **1Y51A (**87, 1.65); **1Z0NA (**87, 1.49); **1Q7LB (**88, 1.40); **1EHDA (**88, 1.50); **1GXUA (**88, 1.27); **1CYO_ (**88, 1.50); **1HFES (**88, 1.60); **1AY7B (**89, 1.70); **1YD0A (**89, 1.50); **1G8QA (**90, 1.60); **1US6A (**91, 1.65); **1S29A (**92, 1.60); **1N0QA (**92, 1.26); **1DGWY (**92, 1.70); **2C3VA (**92, 1.39); **2COVD (**92, 1.25); **2AXIA (**92, 1.40); **1FK5A (**93, 1.30); **1T1VA (**93, 1.60); **1MKKA (**93, 1.32); **1CY5A (**93, 1.30); **2EWHA (**93, 1.40); **2BL8A (**93, 1.60); **1MOLA (**94, 1.70); **1FLTX (**95, 1.70); **2I3HB (**95, 1.62); **1NLQC (**96, 1.50); **1Z21A (**96, 1.50); **1YPHE (**97, 1.34); **1UHEA (**97, 1.55); **1QGEE (**97, 1.70); **1JO0A (**97, 1.37); **1W2LA (**97, 1.30); **2CWRA (**97, 1.70); **1YN3A (**98, 1.35); **1CQMA (**98, 1.65); **1PLC_ (**99, 1.33); **1LK2B (**99, 1.35); **2CCQA (**99, 1.60); **1QDVA (**99, 1.60); **1R1TB (**99, 1.70); **4UBPA (**100, 1.55); **1W41A (**100, 1.70); **1YLXA (**100, 1.60); **2BRFA (**100, 1.40); **1JOSA (**100, 1.70); **1VKEC (**100, 1.56); **3VUB_ (**101, 1.40); **3EZMA (**101, 1.50); **1RKIA (**101, 1.60); **1Y0HA (**101, 1.60); **1MG4A (**101, 1.50); **2ASKA (**101, 1.55); **1XPPD (**101, 1.60); **1QW2A (**102, 1.50); **1P6ZR (**102, 1.67); **1MY7B (**102, 1.49); **1ZLDA (**102, 1.65); **2J73A (**103, 1.40); **3CHBF (**103, 1.25); **1UB4A (**103, 1.70); **1LYQB (**104, 1.50); **1WS8A (**104, 1.60); **1YQHA (**104, 1.70); **1YGTA (**104, 1.70); **1V70A (**105, 1.30); **1FUS_ (**105, 1.30); **1JR8A (**105, 1.50); **7FD1A (**106, 1.30); **256BA (**106, 1.40); **1EW4A (**106, 1.40); **1XLQA (**106, 1.45); **1SBXA (**106, 1.65); **2FCWA (**106, 1.26); **1SQEB (**106, 1.50);

1OU8A (106, 1.60); **1XSVA (**106, 1.70); **1X0TA (**106, 1.60); **1XAWA (**107, 1.45); **1KAFA (**108, 1.60); **2TRXB (**108, 1.68); **1U5DA (**108, 1.70); **1T92A (**108, 1.60); **1NJHA (**108, 1.70); **1OI0A (**108, 1.50); **1VM9A (**109, 1.48); **1XW3A (**110, 1.65); **1KR4A (**110, 1.40); **1NYCA (**111, 1.40); **1VYIA (**111, 1.50); **2B02A (**111, 1.50); **1DDWA (**111, 1.70); **1KPF_ (**111, 1.50); **1LQ9A (**112, 1.30); **2MCM_ (**112, 1.50); **1IBYA (**112, 1.65); **1N13B (**112, 1.40); **1OSYB (**112, 1.70); **1HXIA (**112, 1.60); **1WJXA (**112, 1.70); **1PZ4A (**113, 1.35); **1IFRA (**113, 1.40); **2GDGA (**114, 1.45); **2IC2B (**114, 1.30); **1QBZB (**114, 1.47); **1S67U (**115, 1.50); **1DLWA (**116, 1.54); **1VFJA (**116, 1.70); **1Q0GA (**117, 1.60); **2B3GA (**117, 1.60); **2INWA (**117, 1.50); **1WLUA (**117, 1.45); **1NC7D (**117, 1.55); **1ZMAA (**118, 1.25); **2CYJA (**118, 1.50); **2AWGA (**118, 1.60); **1F3UA (**118, 1.70); **1NBUA (**118, 1.60); **1XBIA (**118, 1.45); **1V30A (**118, 1.40); **1B2PA (**119, 1.70); **2F9HB (**119, 1.57); **2A7BA (**120, 1.65); **1DLFH (**120, 1.45); **1PBJA (**120, 1.40); **1ECSA (**120, 1.70); **2CKKA (**120, 1.45); **1OW4A (**120, 1.60); **1NU0B (**120, 1.60); **1OKOA (**121, 1.60); **2ITEA (**121, 1.60); **1WDDS (**121, 1.35); **1XKPC (**121, 1.70); **1FLMA (**122, 1.30); **1WHI_ (**122, 1.50); **1WOLA (**122, 1.62); **1U9DA (**122, 1.70); **4UBPB (**122, 1.55); **1R29A (**122, 1.30); **2BWQA (**122, 1.41); **1IJYA (**122, 1.35); **1NKOA (**122, 1.45); **2CUAA (**122, 1.60); **1PMY_ (**123, 1.50); **2ERBA (**123, 1.50); **1BGF_ (**124, 1.45); **2CVLA (**124, 1.65); **1H03P (**125, 1.70); **1QVEB (**125, 1.54); **1GY6A (**125, 1.60); **2HNGA (**125, 1.63); **1A62_ (**125, 1.55); **2ASFA (**125, 1.60); **1EAQB (**125, 1.25); **1H2CA (**125, 1.60); **1OOHA (**126, 1.25); **1YRKA (**126, 1.70); **1TP6A (**126, 1.50); **1Z9LA (**126, 1.70); **1DBFA (**127, 1.30); **1G6GA (**127, 1.60); **1RIE_ (**127, 1.50); **2CJTC (**127, 1.44); **1WN9A (**127, 1.58); **1JB3A (**127, 1.60); **2IPRA (**127, 1.50); **1Z67A (**127, 1.45); **2I7SA (**128, 1.35); **2BKMA (**128, 1.50); **2A4XB (**128, 1.40); **1ZPSB (**128, 1.70); **2HEWF (**128, 1.45); **1J34A (**129, 1.55); **2D48A (**129, 1.65); **1XS0C (**129, 1.58); **1J3AA (**129, 1.60); **1VYKA (**129, 1.49); **1EW0A (**130, 1.40); **1PA7A (**130, 1.45); **3NUL_ (**130, 1.60); **1NEPA (**130, 1.70); **2AALB (**130, 1.65); **2F5GA (**130, 1.70); **2D4PA (**130, 1.70); **1C52_ (**131, 1.28); **1YPHC (**131, 1.34); **1UXZA (**131, 1.40); **1FR2B (**131, 1.60); **2LISA (**131, 1.35); **1UY2A (**131, 1.70); **1WVHA (**132, 1.50); **1YKUA (**132, 1.49); **1UGXA (**133, 1.60); **1OA8D (**133, 1.70); **2AVKA (**133, 1.53); **1VP6A (**133, 1.70); **1M4JA (**133, 1.60); **1GP0A (**133, 1.40); **1DQGA (**134, 1.70); **1U7IA (**134, 1.40); **1E29A (**135, 1.21); **1YPQB (**135, 1.40); **1C1LA (**135, 1.50); **1H32B (**135, 1.50); **1ON2A (**135, 1.61); **1K2XB (**135, 1.65); **1J3WB (**135, 1.50); **1S5UE (**136, 1.70); **2J6AA (**136, 1.70); **2FR5A (**136, 1.48); **1EY4A (**136, 1.60); **1TVGA (**136, 1.60); **1Z1SA (**136, 1.49); **1WCKA (**136, 1.36); **1CCWA (**137, 1.60); **2END_ (**137, 1.45); **2FOJA (**137, 1.60); **1EEXG (**137, 1.70); **1QVYA (**138, 1.60); **1S3CA (**138, 1.25); **2A8NB (**138, 1.60); **1KJLA (**138, 1.40); **1H05A (**138, 1.50); **1VH5A (**138, 1.34); **1W1HC (**138, 1.45); **2HX0A (**138, 1.55); **2BCMA (**138, 1.48); **2GECB (**139, 1.30); **2A4DA (**139, 1.69); **1F46B (**140, 1.50); **1WMZA (**140, 1.70); **1LO7A (**140, 1.50); **1GMUC (**140, 1.50); **2JEKA (**140, 1.38); **1TZVA (**141, 1.35); **2D59A (**141, 1.65); **1J30A (**141, 1.70); **1Y2TA (**142, 1.50); **1SJWA (**142, 1.35); **1TFE_ (**142, 1.70); **1M45A (**142, 1.65); **1FM0E (**142, 1.45); **2J1VA (**142, 1.45); **1H6HA (**143, 1.70); **1WMQB (**143, 1.60); **1F2TB (**143, 1.60); **1MJHA (**143, 1.70); **1EYHA (**144, 1.56); **1LJ9A (**144, 1.60); **1O8XA (**144, 1.30); **1T82C (**144, 1.70); **1GS9A (**144, 1.70); **2HD9A (**145, 1.35); **1JKEC (**145, 1.55); **3SDHA (**145, 1.40); **2BSWA (**145, 1.63); **1GVJB (**146, 1.53); **1IT2A (**146, 1.60); **1M9XC (**146, 1.70); **2F3YA (**146, 1.45); **1ID0A (**146, 1.60); **1ZKRA (**146, 1.64); **1ZCEA (**146, 1.30); **1W4SA (**146, 1.55); **1DK8A (**147, 1.57); **2FL4A (**147, 1.60); **2GMYA (**147, 1.60); **1SMBA (**147, 1.55); **1SZHA (**147, 1.50); **1IDPA (**147, 1.45); **2FP7B (**147, 1.68); **1NG6A (**148, 1.40); **1WWIA (**148, 1.58); **1Q1FA (**148, 1.50); **1DZKA (**148, 1.48); **1U69C (**148, 1.60); **2F0CB (**148, 1.65); **1F2UA (**149, 1.60); **1JNRB (**149, 1.60); **1X91A (**149, 1.50); **2BJNB (**149, 1.70); **1NOGA (**149, 1.55); **1DG6A (**149, 1.30); **1IV3A (**150, 1.52); **1WDVA (**150, 1.70); **2B06A (**150, 1.40); **1V4PA (**151, 1.45); **2H30A (**151, 1.60); **1PWBA (**151, 1.40); **1NTVA (**152, 1.50); **1X3KA (**152, 1.64); **2NVHA (**152, 1.53); **1JL1A (**152, 1.30); **1UZKA (**152, 1.35);

1GNYA (153, 1.63); **1SH8A (**153, 1.50); **1E30A (**153, 1.50); **1ELKA (**153, 1.50); **1PINA (**153, 1.35); **2A13A (**153, 1.32); **1SIXA (**153, 1.30); **1HZTA (**153, 1.45); **2FBNA (**153, 1.63); **1HQKA (**154, 1.60); **2F23A (**154, 1.60); **1I12A (**154, 1.30); **1F3UF (**154, 1.70); **1XFSA (**154, 1.70); **1ROCA (**155, 1.50); **1NS5B (**155, 1.68); **1SZ3A (**155, 1.60); **1N08B (**155, 1.60); **1Z9NA (**155, 1.50); **1K2XA (**155, 1.65); **1DWKA (**156, 1.65); **3PVIA (**156, 1.59); **1NZIB (**156, 1.50); **1MXIA (**156, 1.70); **1NYKA (**156, 1.31); **1WRMA (**156, 1.50); **1E7LA (**157, 1.32); **1G1TA (**157, 1.50); **1XWWA (**157, 1.63); **1MK4A (**157, 1.70); **1QJCA (**157, 1.64); **2GRRA (**157, 1.30); **1VHH_ (**157, 1.70); **1RYLA (**157, 1.60); **2GRRB (**157, 1.30); **1RUTX (**157, 1.30); **1Q0NA (**158, 1.25); **1UWFA (**158, 1.69); **1HTWA (**158, 1.70); **2IMJA (**158, 1.50); **2O6CA (**158, 1.70); **1D7PM (**159, 1.50); **1RA9_ (**159, 1.55); **1FJJA (**159, 1.66); **1ZD7A (**159, 1.70); **2FA1B (**159, 1.70); **2HA8A (**159, 1.60); **2I6CA (**160, 1.30); **1RYAA (**160, 1.30); **1QSTA (**160, 1.70); **1R8SA (**160, 1.46); **1ZKKA (**160, 1.45); **1JVWA (**160, 1.70); **2FPRB (**160, 1.70); **1HD2A (**161, 1.50); **1JHJA (**161, 1.60); **1JBOA (**162, 1.45); **2CFEA (**162, 1.50); **1MTYG (**162, 1.70); **1WVQA (**162, 1.45); **1ZXUA (**162, 1.70); **2IKBA (**163, 1.70); **2IMSA (**163, 1.48); **1P36A (**164, 1.45); **2AENB (**164, 1.60); **2DC4A (**164, 1.65); **1FL0A (**164, 1.50); **2B5GA (**164, 1.70); **2G2UB (**165, 1.60); **1K94A (**165, 1.70); **1XEOA (**165, 1.30); **1V8CA (**165, 1.60); **2BSCA (**165, 1.40); **2F6LA (**166, 1.70); **1Z6NA (**166, 1.50); **2BDRA (**166, 1.60); **1IO0A (**166, 1.45); **1G12A (**167, 1.60); **2A50B (**167, 1.30); **1I0RB (**168, 1.50); **1WKCA (**168, 1.70); **1NWAA (**168, 1.50); **1YN9B (**169, 1.50); **1U14A (**169, 1.68); **1JLJA (**169, 1.60); **2BEMA (**170, 1.55); **1TYJA (**170, 1.60); **2IA1B (**170, 1.59); **2FYQA (**170, 1.50); **1Y43B (**171, 1.40); **2A2KA (**171, 1.52); **1OQVA (**171, 1.30); **1NQZA (**171, 1.70); **2FSRA (**171, 1.52); **1KOE_ (**172, 1.50); **1YE8A (**172, 1.40); **2CIHA (**172, 1.50); **1DMGA (**172, 1.70); **1SXRA (**173, 1.56); **1LQVB (**173, 1.60); **1OH4A (**174, 1.35); **2BRJA (**174, 1.50); **1RTTA (**174, 1.28); **2BZ1A (**174, 1.54); **1QFTA (**175, 1.25); **1RXQB (**175, 1.70); **2EW0A (**175, 1.40); **1S0PA (**176, 1.40); **1QGWD (**176, 1.63); **1WUBA (**176, 1.65); **1NG2A (**176, 1.70); **1K1EA (**177, 1.67); **1D4OA (**177, 1.21); **1JFUB (**177, 1.60); **1USCA (**178, 1.24); **1DGWA (**178, 1.70); **1G2QA (**178, 1.50); **1EEXB (**178, 1.70); **1IM5A (**179, 1.65); **1ISPA (**179, 1.30); **2ACFD (**179, 1.40); **2IYVA (**179, 1.35); **2FD5A (**180, 1.70); **1WNYA (**180, 1.60); **2IU5A (**180, 1.60); **1M4IA (**181, 1.50); **1SHUX (**181, 1.50); **2J8KA (**181, 1.50); **1YTQA (**181, 1.70); **1YPYA (**182, 1.51); **2J12A (**182, 1.50); **2J2JA (**182, 1.50); **1PKHA (**182, 1.42); **2HIYD (**183, 1.40); **2FHPA (**183, 1.60); **1K4NA (**183, 1.60); **2C2QA (**183, 1.70); **1PVMB (**184, 1.50); **1UEBA (**184, 1.65); **2CXXA (**184, 1.70); **1UWWA (**184, 1.40); **153L_ (**185, 1.60); **1H6FB (**186, 1.70); **2B5HA (**186, 1.50); **1S99A (**186, 1.65); **1ZR3B (**186, 1.66); **1WPNA (**187, 1.30); **2FI1A (**187, 1.40); **1SK7A (**187, 1.60); **2GENA (**188, 1.70); **1J2RA (**188, 1.30); **1PZ7A (**188, 1.42); **1TXLA (**188, 1.70); **2J21A (**188, 1.60); **1TUAA (**189, 1.50); **1M70A (**190, 1.25); **1UCDA (**190, 1.30); **1X82A (**190, 1.50); **2DG5B (**190, 1.60); **2CVEA (**190, 1.60); **1LKOA (**190, 1.63); **2G7SA (**190, 1.40); **1S8NA (**190, 1.48); **1V2XA (**191, 1.50); **1G3P_ (**191, 1.46); **1Q7LA (**192, 1.40); **1UI0A (**192, 1.50); **1JUVA (**193, 1.70); **1O7NB (**193, 1.40); **1M55A (**193, 1.40); **2GS5A (**193, 1.50); **1MF7A (**194, 1.25); **2NX4A (**194, 1.70); **2NR7A (**194, 1.30); **1NXMA (**194, 1.30); **2BNMA (**194, 1.70); **1PP0B (**194, 1.42); **2COHA (**194, 1.50); **2CZ1A (**194, 1.39); **1Q92A (**195, 1.40); **1QMVA (**195, 1.70); **1YLLD (**195, 1.64); **1NKR_ (**195, 1.70); **1R8ME (**195, 1.70); **2ETXB (**195, 1.33); **1TX4A (**196, 1.65); **2CCJA (**197, 1.70); **1CUJ_ (**197, 1.60); **2HXIA (**197, 1.70); **1OCYA (**198, 1.50); **1T5BA (**199, 1.40); **2GWMA (**200, 1.50); **1D02B (**200, 1.70); **1W0HA (**200, 1.59); **1NOX_ (**200, 1.59); **2GZ4A (**200, 1.50); **1OGAD (**200, 1.40); **1OMRA (**201, 1.50); **2ET1A (**201, 1.60); **1KUFA (**201, 1.35); **1R45A (**201, 1.57); **2NNUA (**201, 1.59); **1S9UA (**201, 1.38); **1NLNA (**203, 1.60); **1OI6B (**203, 1.40); **2CJLA (**204, 1.50); **1WBEA (**204, 1.36); **1W3OA (**204, 1.60); **2IUWA (**204, 1.50); **1HD5A (**205, 1.70); **2HFT_ (**205, 1.69); **1K7JA (**206, 1.40); **1RKUA (**206, 1.47); **1IU8A (**206, 1.60); **1E4CP (**206, 1.66); **1NF9A (**207, 1.50); **1L6XA (**207, 1.65); **2G7EA (**207, 1.60); **1IW0A (**207,

1.40); **2O6SA (**208, 1.50); **1UYLA (**208, 1.40); **1GK9A (**208, 1.30); **2ERFA (**209, 1.45); **1JKXA (**209, 1.60); **1EU3A (**210, 1.68); **1L7MB (**210, 1.48); **1E9EA (**210, 1.60); **1RO2A (**210, 1.60); **2HJEA (**210, 1.70); **2ICUA (**210, 1.60); **1N0WA (**210, 1.70); **2B82A (**211, 1.25); **1FT5A (**211, 1.60); **2CZ1B (**211, 1.39); **1MJUH (**211, 1.22); **2HALA (**212, 1.35); **1JAYA (**212, 1.65); **1O8BB (**212, 1.25); **1ZD8A (**212, 1.48); **1ES9A (**212, 1.30); **1YUMA (**212, 1.70); **2BIVA (**212, 1.70); **1KM4A (**212, 1.50); **1WBHB (**214, 1.55); **2AYH_ (**214, 1.60); **1AZO_ (**214, 1.70); **1RZ2A (**214, 1.60); **2CL5A (**215, 1.60); **2F8YA (**215, 1.55); **1Z72A (**216, 1.45); **1VHQA (**216, 1.65); **1PG6A (**216, 1.70); **2G19A (**216, 1.70); **2O8LA (**216, 1.50); **1C1KA (**217, 1.45); **1JFXA (**217, 1.65); **2BO9B (**217, 1.60); **1V8EA (**217, 1.50); **1GPPA (**217, 1.35); **1MJUL (**219, 1.22); **2J43A (**219, 1.60); **2AVDA (**219, 1.70); **1KGSA (**219, 1.50); **1XTAA (**220, 1.58); **1U9CA (**220, 1.35); **1H99A (**220, 1.55); **1K3YA (**221, 1.30); **1EUVA (**221, 1.60); **1MQOA (**221, 1.35); **1O26B (**221, 1.60); **2FT0A (**222, 1.66); **2NTUA (**222, 1.53); **2HXMA (**223, 1.30); **1DFMA (**223, 1.50); **1OQ1A (**223, 1.70); **1U5HA (**223, 1.65); **1TKEA (**224, 1.46); **1DAD_ (**224, 1.60); **1I1NA (**224, 1.50); **1T61D (**224, 1.50); **1L6RA (**225, 1.40); **1G61A (**225, 1.30); **2CULA (**225, 1.65); **1L3LC (**225, 1.66); **1W2YA (**226, 1.65); **2ASBA (**226, 1.50); **2TPSB (**227, 1.25); **1G8AA (**227, 1.40); **1JE0C (**227, 1.60); **2B4HA (**227, 1.60); **1FJ2A (**229, 1.50); **2BZGA (**229, 1.58); **1V58A (**229, 1.70); **1JYKA (**229, 1.50); **2CXAA (**229, 1.60); **1YXYA (**230, 1.60); **2F6UA (**231, 1.55); **2CBZA (**231, 1.50); **1SQSA (**232, 1.50); **2BKAA (**232, 1.70); **2FSQA (**232, 1.40); **2AXCA (**232, 1.70); **1ZI8A (**233, 1.40); **2C5QE (**233, 1.70); **2AGKA (**233, 1.30); **1M65A (**234, 1.57); **1QWYA (**234, 1.30); **1WQWB (**235, 1.45); **1OOEA (**235, 1.65); **1TWDB (**235, 1.70); **1QB7A (**236, 1.50); **1Z3XA (**236, 1.50); **1K0MB (**236, 1.40); **1FJHA (**236, 1.68); **1UFOA (**238, 1.60); **1XDZA (**238, 1.60); **1YCDB (**238, 1.70); **1QG8A (**238, 1.50); **2C3NA (**239, 1.50); **1E59A (**239, 1.30); **1F5VA (**240, 1.70); **1V00A (**240, 1.70); **2BR6A (**240, 1.70); **1TZPA (**240, 1.40); **1B5EA (**241, 1.60); **2IBNA (**241, 1.50); **1T0BH (**241, 1.70); **2BKXA (**242, 1.40); **1UD9A (**242, 1.68); **1RV9A (**242, 1.53); **1MVEA (**243, 1.70); **2APJA (**243, 1.60); **1UJPA (**243, 1.34); **1NQ7A (**244, 1.50); **1K55A (**244, 1.39); **1WCV1 (**244, 1.60); **1AH7_ (**245, 1.50); **2F2BA (**245, 1.68); **1ZVTB (**246, 1.70); **2H8GA (**246, 1.50); **1KYFA (**247, 1.22); **2FZVB (**247, 1.70); **1M2KA (**249, 1.47); **1O9GA (**249, 1.50); **1V6TA (**249, 1.70); **2GZSA (**249, 1.40); **2BSYA (**249, 1.50); **1TWYA (**249, 1.65); **1SG4C (**250, 1.30); **1T7RA (**250, 1.40); **2DQWA (**250, 1.65); **1V9FA (**250, 1.70); **1E2WA (**251, 1.60); **1LV7A (**251, 1.50); **1R5LA (**251, 1.50); **1ZKPA (**251, 1.50); **2O3TA (**252, 1.68); **1THFD (**253, 1.45); **1ZS9A (**253, 1.70); **1KZQA (**253, 1.70); **2I53A (**254, 1.50); **1WCWA (**254, 1.30); **1K3WA (**254, 1.42); **2IWAA (**254, 1.60); **1EKQB (**254, 1.50); **1P99A (**255, 1.70); **1YB6A (**256, 1.54); **1V7ZA (**257, 1.60); **2A14A (**257, 1.70); **2BGIA (**257, 1.68); **1GS5A (**258, 1.50); **1MQDA (**258, 1.46); **1OAA_ (**259, 1.25); **1QGIA (**259, 1.60); **1QH5A (**260, 1.45); **1K77A (**260, 1.63); **2CCHB (**260, 1.70); **1ES5A (**260, 1.40); **1H32A (**261, 1.50); **1J31A (**262, 1.60); **2CKIA (**262, 1.70); **1K07A (**262, 1.65); **2GNPA (**262, 1.65); **1UQ5A (**263, 1.40); **2G8OB (**263, 1.30); **1LZJA (**263, 1.32); **1DJ0A (**264, 1.50); **2DEKA (**265, 1.65); **1O9IA (**266, 1.33); **2NW8A (**266, 1.60); **2GQTA (**267, 1.30); **1AKO_ (**268, 1.70); **2H7MA (**268, 1.62); **1UEKA (**268, 1.70); **1CB0A (**268, 1.70); **2FY7A (**268, 1.70); **1JOVA (**269, 1.57); **2CZLA (**269, 1.55); **1OI7A (**270, 1.23); **1O4YA (**270, 1.48); **1NYTA (**271, 1.50); **1RKQA (**271, 1.40); **1W5RA (**273, 1.45); **1OJRA (**274, 1.35); **2GKEA (**274, 1.35); **1LK2A (**274, 1.35); **1TA3A (**274, 1.70); **2CITA (**274, 1.40); **2AIJX (**274, 1.55); **1LTZA (**274, 1.40); **1DCIA (**275, 1.50); **2BJIA (**275, 1.30); **2B9EA (**275, 1.65); **1QQFA (**276, 1.45); **2F1KA (**277, 1.55); **2A84A (**277, 1.55); **1I60A (**278, 1.60); **1RP0A (**278, 1.60); **1XUBA (**278, 1.30); **1JTVA (**278, 1.54); **1N57A (**279, 1.60); **1DQZA (**280, 1.50); **1SI6X (**280, 1.45); **1B8OA (**280, 1.50); **2FN9A (**280, 1.40); **1AQUA (**281, 1.60); **1QV9A (**282, 1.54); **1U0KB (**282, 1.50); **1WB4A (**283, 1.40); **1QXMA (**283, 1.70); **1LYVA (**283, 1.36); **1YNPA (**283, 1.25); **2CWCA (**284, 1.65); **2CM2A (**284, 1.50); **1FIUA (**286, 1.60); **1ZXIC (**287, 1.70); **1XG4A (**287, 1.60); **2CIRA (**287,

1.60); **1Q74A (**287, 1.70); **1T9HA (**287, 1.60); **2DPLA (**287, 1.43); **1L9XA (**288, 1.60); **1X2JA (**290, 1.60); **1P0HA (**290, 1.60); **1H6TA (**291, 1.60); **1UV4A (**291, 1.50); **1BN6A (**291, 1.50); **1F74A (**293, 1.60); **1FXOB (**293, 1.66); **1RHS_ (**293, 1.36); **1OFWA (**293, 1.50); **1INLC (**293, 1.50); **1VPDA (**294, 1.65); **1WRRA (**294, 1.64); **1A4IB (**295, 1.50); **2BMWA (**295, 1.50); **2GB7B (**295, 1.70); **2FAOA (**295, 1.50); **1YI9A (**295, 1.70); **1FTRA (**296, 1.70); **1Q0RA (**297, 1.45); **2CL2A (**298, 1.35); **2DDRC (**298, 1.40); **1XKGA (**298, 1.61); **1US5A (**298, 1.50); **1IN4A (**298, 1.60); **2E2OA (**299, 1.65); **2CWLA (**299, 1.65); **1IZCA (**299, 1.70); **2IXMA (**300, 1.50); **1TZCA (**301, 1.45); **1ZDYA (**301, 1.44); **1VE1A (**302, 1.45); **1WRVB (**305, 1.50); **1MLA_ (**305, 1.50); **1NTYA (**305, 1.70); **2H6NB (**305, 1.50); **1T6CA (**306, 1.53); **2CTC_ (**307, 1.40); **2GASA (**307, 1.60); **1GWUA (**307, 1.31); **1Q8FA (**308, 1.70); **1IJQA (**308, 1.50); **1R9LA (**309, 1.59); **1GCA_ (**309, 1.70); **1DMHA (**309, 1.70); **1LTM_ (**309, 1.70); **2DPOA (**310, 1.70); **2GQ1A (**310, 1.45); **1JL0B (**310, 1.50); **1DD9A (**310, 1.60); **1JUBA (**311, 1.40); **1DC1B (**311, 1.70); **2CYGA (**312, 1.45); **1OFZA (**312, 1.50); **1NVMB (**312, 1.70); **1OGQA (**313, 1.70); **1E19A (**313, 1.50); **1Y8AA (**313, 1.40); **1F9VA (**313, 1.30); **2AEBA (**314, 1.29); **1P4OB (**314, 1.50); **1Y9UA (**314, 1.39); **1FCQA (**314, 1.60); **2H6FA (**315, 1.50); **1TJYA (**316, 1.30); **1KEIA (**316, 1.60); **2FQXA (**316, 1.70); **1YIIA (**316, 1.42); **1E2KB (**316, 1.70); **1SVSA (**316, 1.50); **1TCA_ (**317, 1.55); **1LZLA (**317, 1.30); **1S1DA (**317, 1.60); **1L7AA (**318, 1.50); **2D81A (**318, 1.66); **2CB2A (**318, 1.70); **2FFCA (**318, 1.70); **2BOQA (**319, 1.33); **1WZZA (**319, 1.65); **1W5QB (**319, 1.40); **1MTPA (**320, 1.50); **1F1UA (**322, 1.50); **1R6DA (**322, 1.35); **2AFWA (**323, 1.56); **1JSRA (**324, 1.70); **1OZ2A (**324, 1.55); **1WER_ (**324, 1.60); **2FH1B (**324, 1.55); **2FSTX (**324, 1.45); **2DF8A (**325, 1.50); **1QD1B (**325, 1.70); **1S95B (**325, 1.60); **1XGKA (**325, 1.40); **2FMPA (**326, 1.65); **1TBFA (**326, 1.30); **2C7PA (**327, 1.70); **2BJFA (**328, 1.67); **1WMWA (**328, 1.55); **2J0PA (**328, 1.70); **1YDYA (**328, 1.70); **1YG9A (**329, 1.30); **1OBNA (**329, 1.30); **2C6QB (**329, 1.70); **1Q11A (**329, 1.60); **2FGQX (**330, 1.45); **2HBVB (**330, 1.65); **1YLEA (**330, 1.70); **1FO8A (**330, 1.40); **1I0DA (**331, 1.30); **1GXMB (**332, 1.32); **2BFDB (**332, 1.39); **2DBQA (**333, 1.70); **1OBFO (**334, 1.70); **1TXGA (**335, 1.70); **1NE9A (**335, 1.70); **1Y0YA (**335, 1.60); **1CZFA (**335, 1.68); **2AEXA (**336, 1.58); **1PBYB (**337, 1.70); **1KRHA (**337, 1.50); **1WTJB (**337, 1.55); **1XH8A (**337, 1.60); **1JX6A (**338, 1.50); **1J79B (**338, 1.70); **1NVMG (**339, 1.70); **1MXRA (**339, 1.42); **1X7DA (**340, 1.60); **1Y1PA (**342, 1.60); **1XFIA (**343, 1.70); **1QNRA (**344, 1.40); **1FP2A (**345, 1.40); **1USGA (**346, 1.53); **1PXZA (**346, 1.70); **2NQTA (**346, 1.58); **1EZWA (**347, 1.65); **2DE3B (**347, 1.60); **1C1DA (**349, 1.25); **1JIXA (**351, 1.65); **2DG5A (**351, 1.60); **1R5MA (**351, 1.55); **1MG7B (**352, 1.55); **2C0HA (**353, 1.60); **1Z6FA (**354, 1.60); **1B6A_ (**355, 1.60); **1XSZA (**356, 1.41); **1RCQA (**356, 1.45); **1R3SA (**356, 1.65); **1IUQA (**357, 1.55); **2B61A (**357, 1.65); **1DOSA (**358, 1.67); **2ARRA (**358, 1.55); **2H8ZA (**359, 1.42); **1QCXA (**359, 1.70); **2CDCA (**359, 1.50); **1YQDA (**359, 1.65); **2A9DB (**359, 1.70); **1RGYA (**360, 1.52); **2IURA (**360, 1.30); **1R0MA (**360, 1.30); **2FXUA (**360, 1.35); **1UASA (**362, 1.50); **2F26A (**362, 1.58); **1GC0C (**362, 1.70); **1NC5A (**363, 1.60); **1XRFA (**363, 1.65); **1GU7A (**364, 1.70); **1KQ3A (**364, 1.50); **2C5AA (**364, 1.40); **1VKPA (**365, 1.53); **1OK7A (**366, 1.65); **1Z8GA (**366, 1.55); **1HZ4A (**366, 1.45); **2AEUA (**366, 1.70); **1T4BA (**367, 1.60); **1ZZ1A (**367, 1.57); **1URSA (**367, 1.45); **1P0BA (**369, 1.70); **1WU4A (**374, 1.35); **2BFDA (**374, 1.39); **1YT3A (**375, 1.60); **1ONWA (**375, 1.65); **1YU0A (**376, 1.56); **2I71B (**376, 1.70); **2AHFA (**377, 1.52); **1SG6B (**378, 1.70); **1ZY7A (**378, 1.70); **1EDG_ (**380, 1.60); **2GSOA (**382, 1.30); **1NOFA (**383, 1.42); **1BS0A (**383, 1.65); **2C1IA (**383, 1.35); **1MTYB (**384, 1.70); **2DCFA (**384, 1.40); **1V5DA (**386, 1.50); **2D29A (**386, 1.65); **1R6XA (**386, 1.40); **1F8EA (**388, 1.40); **1I88A (**388, 1.45); **1V6SA (**390, 1.50); **1QOPB (**390, 1.40); **2B2HA (**391, 1.54); **1M3KA (**392, 1.70); **2B3FA (**392, 1.56); **1KOLA (**396, 1.65); **2C78A (**397, 1.40); **1HFEL (**397, 1.60); **2I49A (**398, 1.35); **1V5VA (**399, 1.50); **1F24A (**399, 1.40); **1RU4A (**400, 1.60); **1JNDA (**400, 1.30); **2BWRA (**401, 1.50); **1A12A (**401, 1.70); **1D8WA (**402, 1.60); **1QS1A (**402, 1.50); **2DBNA (**403, 1.70);

1HT6A (404, 1.50); **1P1MA (**404, 1.50); **1XK7A (**407, 1.60); **1S9RA (**409, 1.60); **2H6FB (**410, 1.50); **1AJSA (**412, 1.60); **1OX0A (**414, 1.30); **1D0CA (**416, 1.65); **1YIZA (**417, 1.55); **1EJDA (**418, 1.55); **1HYOB (**419, 1.30); **1GSOA (**419, 1.60); **1HQSA (**422, 1.55); **1M7YA (**424, 1.60); **1G6SA (**427, 1.50); **4UAGA (**428, 1.66); **1GPIA (**430, 1.32); **1VCLA (**431, 1.70); **2C61A (**432, 1.50); **1KS8A (**433, 1.40); **1X54A (**434, 1.45); **1KAEA (**434, 1.70); **2HXTA (**434, 1.70); **1CSH_ (**435, 1.60); **1QWOA (**435, 1.50); **2AL1A (**436, 1.50); **1ZHXA (**436, 1.50); **1Y9ZB (**436, 1.40); **1YQZA (**437, 1.54); **1TUOA (**437, 1.70); **2BWVA (**439, 1.70); **1PMI_ (**440, 1.70); **2BCGG (**442, 1.48); **2AXQA (**445, 1.70); **1Y6VA (**449, 1.60); **1A8D_ (**452, 1.57); **1CRUB (**452, 1.50); **1CJCA (**455, 1.70); **2EX2A (**456, 1.55); **1AOP_ (**456, 1.60); **1GKPA (**457, 1.29); **1ZO4B (**457, 1.46); **3GRS_ (**461, 1.54); **1K7IA (**462, 1.59); **2ICYB (**463, 1.64); **1KV7A (**463, 1.40); **1O6VA (**464, 1.50); **1WDDA (**464, 1.35); **1PO5A (**465, 1.60); **2JC9A (**467, 1.50); **1OOYB (**468, 1.70); **1WLEB (**469, 1.65); **1FS7A (**471, 1.60); **2GUYA (**476, 1.59); **1ZR6A (**479, 1.55); **1W1OA (**479, 1.70); **1OFLA (**480, 1.70); **1CCWB (**483, 1.60); **1LAM_ (**484, 1.60); **1WZAA (**488, 1.60); **1PBYA (**489, 1.70); **1U8VA (**490, 1.60); **1J1NA (**492, 1.60); **1LJ8A (**492, 1.70); **1WDPA (**493, 1.27); **2FFUA (**494, 1.64); **1S3EB (**494, 1.60); **1Z32X (**495, 1.60); **1V0WA (**496, 1.35); **1E6PB (**497, 1.70); **1IJHA (**498, 1.53); **1HFUA (**500, 1.68); **1NKGA (**508, 1.50); **1O98A (**509, 1.40); **2A65A (**509, 1.65); **1MTYD (**512, 1.70); **1QMGA (**514, 1.60); **1WVFA (**515, 1.30); **2BJKA (**516, 1.40); **1HP1A (**516, 1.70); **1JU2A (**521, 1.47); **1P1JA (**525, 1.70); **1GX5A (**530, 1.70); **1W4XA (**533, 1.70); **1LLFA (**534, 1.40); **1Y7BA (**534, 1.60); **1EDQA (**540, 1.55); **1I19A (**541, 1.70); **1GNLA (**543, 1.25); **1KDGB (**546, 1.50); **1EEXA (**551, 1.70); **1MW9X (**556, 1.67); **1GK9B (**557, 1.30); **2CXNA (**557, 1.40); **1Q8AA (**559, 1.70); **1QKSA (**559, 1.28); **1Y0PA (**568, 1.50); **4UBPC (**569, 1.55); **1F5NA (**570, 1.70); **1OGOX (**572, 1.65); **2F5VA (**577, 1.41); **1U4BA (**580, 1.60); **1V5EA (**590, 1.60); **1FWXA (**591, 1.60); **1T1UA (**597, 1.55); **1W8OA (**601, 1.70); **1X38A (**602, 1.70); **2D5WA (**602, 1.30); **1QSAA (**618, 1.65); **1W6GA (**619, 1.55); **1S0IA (**623, 1.60); **1JG9A (**628, 1.66); **1JI1A (**637, 1.60); **1GOF_ (**639, 1.70); **1JNRA (**642, 1.60); **1KWGA (**644, 1.60); **1PN0C (**656, 1.70); **1KB0A (**669, 1.44); **1QHOA (**686, 1.70); **1W27A (**689, 1.70); **1GQIA (**708, 1.48); **1H2WA (**710, 1.39); **1RKYA (**734, 1.68); **1W0PA (**753, 1.60); **1RWHA (**754, 1.25); **1H16A (**759, 1.53); **1V7WA (**779, 1.60); **1G8KA (**822, 1.64); **1YGE_ (**839, 1.40); **2DQ6A (**865, 1.50); **1VLBA (**907, 1.28); **1KQFA (**981, 1.60); **1JZ7A (**1010, 1.50); **1GTED (**1014, 1.65); **2FHFA (**1052, 1.65).
